# Effects of Elaboration and Instructor Feedback on Retention of Clinical Reasoning Competence Among Undergraduate Medical Students: A Randomized Crossover Trial

**DOI:** 10.1001/jamanetworkopen.2022.45491

**Published:** 2022-12-06

**Authors:** Milena Berens, Tim Becker, Sven Anders, Amir H. Sam, Tobias Raupach

**Affiliations:** 1 Department of Cardiology and Pneumology, Göttingen University Medical Centre, Göttingen, Germany; 2 Study Deanery, University Medical Centre Göttingen, Göttingen, Germany; 3 Department of Legal Medicine, University Medical Centre Hamburg-Eppendorf, Hamburg, Germany; 4 Medical Education Research Unit, Imperial College School of Medicine, Imperial College London, London, United Kingdom; 5 Institute of Medical Education, Medical Faculty, University of Bonn, Germany

## Abstract

This randomized crossover trial examines whether elaboration on common errors in patient treatment, combined with individualized mailed feedback, improves medium-term retention of clinical reasoning competence.

## Introduction

Diagnostic errors can lead to significant patient harm,^1^ and educational interventions may help prevent them. One such educational intervention, repeated testing with case-based key feature questions during which students actively suggest the most appropriate next step (the *key feature*) in the care of a virtual patient, warrants study.^2^ Adding elaboration and instructor feedback specifically focusing on common misconceptions^3^ may have a greater effect on learning than repeated testing alone. We studied whether elaboration on common errors in patient care, combined with individualized mailed feedback, improves medium-term retention of clinical reasoning competence.

## Methods

This crossover trial was approved by the Universitätsmedizin Göttingen ethics committee. Participants provided written informed consent. This study was registered on ClinicalTrials.gov (NCT05585892). The trial protocol and statistical analysis plan are provided in Supplement 1. This study is reported following the Consolidated Standards of Reporting Trials (CONSORT) reporting guideline.

From winter 2018 until summer 2019, fourth-year undergraduate medical students participated in a prospective randomized crossover trial with a 6-month follow-up (eFigure in Supplement 2). In parallel to attending formal teaching on general medicine, they attended 10 weekly digital key feature examinations (e-seminars). There were 2 student groups: in one group, half of the questions were shown as long-menu format key feature questions (control items). The other half (intervention items) also consisted of such key feature questions but were followed by a short written elaboration task prompting students to differentiate the correct answer from a common clinical reasoning error identified in the responses from previous student cohorts. Following each e-seminar, students received individual emails containing their own answers and expert comments (Table). In the second group, the assignment of intervention and control items was opposite to that in the first group. At the end of term (exit examination) as well as 6 months later (retention test), students took formative examinations covering the same content. The primary outcome was the within-participant difference in percentage scores in intervention vs control items in the retention test, assessed using 2-sided paired *t* tests with α = .05. Further details are provided in the eMethods in Supplement 2. Statistical analyses were performed using SPSS software version 26.0 (IBM). Data were analyzed from August 2021 to January 2022.

**Table.** Example of a Key Feature Item With Elaboration Question, Grading Rubric for Free-Text Answers, and Excerpt From an Original Feedback Email

Element	Example
Case vignette	Female patient aged 44 y, smoker with back pain, immobilization for 7 d, and sudden shortness of breath following defecation; hypotension and tachycardia
Key feature question	What is the most likely diagnosis?
Potential answers	
Correct answer	Acute pulmonary embolism
Target incorrect answer	Aortic dissection or myocardial infarction
Elaboration question	Why is the diagnosis of acute pulmonary embolism more likely in this patient than an aortic dissection or a myocardial infarction?
Grading for free-text answers	
Completely correct answer (3 points)	“Immobilization may have given rise to thromboembolism; no pain radiation as in aortic dissection or myocardial infarction.”
Partially correct answer (2 points)	“Immobilization and subsequent thromboembolism upon using the toilet.”
Insufficient answer (1 point)	”Smoking and lack of exercise.”
Slack answer (0 points)	Do not know.
Feedback email (excerpt)	“The most likely diagnosis of the 44 year old patient with back pain and sudden shortness of breath was an acute pulmonary embolism – given that she had been immobilized for a few days. We asked you to explain why this diagnosis was more likely than an aortic dissection or a myocardial infarction. Here’s our expert comment: ”The risk factors emerging from the patient history (immobilization, smoking) as well as clinical signs and symptoms (shortness of breath) suggest acute pulmonary embolism (PE). This can be triggered (among others) by getting up in the morning, defecation or sudden strenuous activity. “One important differential diagnosis is acute coronary syndrome (ACS) which, by definition, is characterized by chest pain. However, differentiating acute PE from ACS can be difficult, and many PE cases are still not diagnosed. “Acute aortic dissection is also associated with pain – however, this is often described as a tearing/ripping sensation, spreading to the neck or down the back. One characteristic finding in this disease is a difference in blood pressure measurements between the left and the right arm. Aortic dissection is much less common than the other two diagnoses. “In case of the patient described in the e-seminar, both the immobilization and the association between the visit to the toilet and symptom onset are indicative of acute PE. “Please see for yourself if you have listed all relevant aspects in your own answer: “‘Lack of activity due to back pain plus smoking increase the risk of thromboembolism – hence the suspicion of pulmonary embolism.’”

## Results

A total of 83 fourth-year undergraduate medical students (mean [SD] age, 25.0 [3.9] years; 51 [61%] women; mean [SD] score in summative examinations in the preceding term, 80.8% [6.3 percentage points]) participated in the trial. In both the exit examination and the retention test, students performed significantly better on intervention than on control items (mean [SD] score: exit examination, 71.3% [19.8 percentage points] vs 66.4% [21.3 percentage points]; *P* = .003; Cohen *d* = 0.33; retention test, 67.1% [20.8 percentage points] vs 62.9% [21.6 percentage points]; *P* = .009; Cohen *d* = 0.29). This effect was driven by significant reductions in the frequency of common errors in clinical reasoning (Figure). A multivariate regression analysis with retention test performance as the dependent variable and previous examination performance, student age and gender, scores in key feature questions during e-seminars, and scores in elaboration questions as independent variables found that a higher percentage of complete written answers to elaboration questions were associated with better performance on intervention items in the retention test, whereas previous performance in examinations was not.

**Figure.**
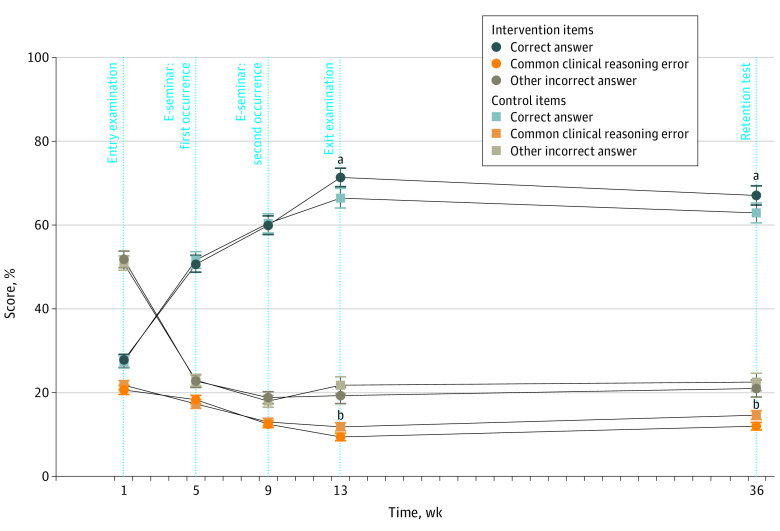
Student Performance in Intervention and Control Items Throughout the Randomized Trial Error bars indicate SEs of the mean. ^a^*P* < .01 for paired *t* tests. ^b^*P* < .05.

## Discussion

This randomized crossover trial found a significant effect of a simple educational intervention on clinical reasoning competence. The fact that the improvement in performance in intervention items was mirrored by a reduction in common clinical reasoning errors suggests a causal link between the intervention and the educational effect. The intervention worked equally well regardless of previous examination scores and was still significant at 6 months, unlike in previous studies.^4,5^ The prospective crossover design by which each student was exposed to the intervention as well as the control format is one strength of this work. The 2 main limitations of this trial are its single-center design and the lack of patient outcome data. Additional research should be conducted to test whether the intervention will lead to recognition and reduction of errors in clinical practice.

## Notes

Supplement 1.
Trial Protocol and Statistical Analysis Plan

Supplement 2. **eFigure.** CONSORT 2010 Flow Diagram
eMethods.

Supplement 3.
Data Sharing Statement
