# Supplementary material for: Effects of Elaboration and Instructor Feedback on Retention of Clinical Reasoning Competence Among Undergraduate Medical Students: A Randomized Crossover Trial
Source: JAMA Netw Open. 2022 Dec 6;5(12):e2245491. doi: 10.1001/jamanetworkopen.2022.45491 (PMC9856325; doi:10.1001/jamanetworkopen.2022.45491)
Supplement: Supplement 1. — Trial Protocol and Statistical Analysis Plan [file jamanetwopen-e2245491-s001.pdf]

# Trial Protocol

# Title:

# „Randomised cross-over Study on the Effectiveness of Elaboration and Proactive Feedback as Part of Formative Key Feature Examinations in Undergraduate Medical Education“

Project Lead:
Prof. Dr. med. T. Raupach
Division of Medical Education Research and Curriculum Development
University Medical Centre Göttingen

**There is no third-party funding or support for this trial.**

## 1. Study aim

Repeated formative (i.e., non-graded) testing enhances student learning outcome on clinical reasoning skills. [1, 2]. At University Medical Centre Göttingen (UMG), a number of trials investigating the so-called *testing effect* have already been conducted in the past. They showed, amongst others, that dealing with videotaped clinical cases compared with written cases increases short-term outcome but not long-term retention [3]. More recently, one study addressed the question whether clinical reasoning skills can be fostered by an elaboration of incorrect answers. Results of a previous trial had suggested that a considerable number of students were not sufficiently motivated to provide thorough answers to elaboration questions. This impression remained even after introducing financial incentives for students although a small but significant effect of the intervention was noted (percent score in the exit exam: 65.7 ± 19.6% vs. 62.3 ± 22.9%; $p = 0.022$). Yet, student performance remained moderate at best. Thus, the intervention will now be extended by including automated feedback provided by email. All students participating in an electronic case-based seminar (e-seminar) will receive an individual email after the event, displaying their raw point score as well as their written answers to elaboration questions and expert comments reflecting current medical knowledge.

This trial addresses the following research question:

*What is the effect of elaboration and consecutive automated and individual feedback following e-seminars on medical students' clinical reasoning skills?*

## 2. Background and previous work

According to recent findings [4], retrieval of knowledge is not a passive process. Instead, long-term retention is being facilitated by the act of retrieval itself (‚retrieval hypothesis'). Potentially, this effect that has also been called 'direct testing effect', could also be due to additional exposure to the content during an assessment [5]. However, complex studies in which exposure was experimentally controlled did not lend support to this 'total time hypothesis' [6]. The effectiveness of examinations as memory boosters with respect to medical education has been shown in a number studies [7, 8]. However, many of these used short follow-up periods (e.g., 7 days) [9] or implemented reproduction tests on a low taxonomic level [10]. Yet, these studies suggest that formative examinations may promote learning processes. According to a review of the topic [11], these exams should contain production tests and be repeated with appropriate spacing. In addition, students should receive feedback shortly after the exam.

Given these recommendations, longitudinal key feature examinations were implemented in three consecutive teaching modules at our institution in 2013. These case-based examinations lend themselves to fostering complex cognitive skills. A key feature is defined as a critical step in solving a clinical problem [12]. According to this definition, a key feature case consists of a case vignette and approximately five consecutive questions relating to the diagnostic and therapeutic approach. In contrast to single-best answer multiple choice questions, students cannot choose from a list of five answer options but must produce a written answer. Thus, rather than recognizing the correct answer, the aim of a key feature examination is to actively produce a correct answer [5]. In order to save students from making follow-on mistakes, they are informed about the correct answers to preceding questions whenever attempting to answer the next question. At this point, students also receive static feedback on their previous answer.

Recently, the results of a randomized cross-over trial comparing active retrieval using key feature questions with repeated study of the same material were published [1]. The data showed that working on key feature cases with static feedback elicited a larger medium-term learning outcome than passive restudying of the same content. The specific role of the feedback in the process however remained unclear.

Current findings from educational psychology research [13] suggest that diagnostic errors made in a protected learning environment can serve as starting points for further elaboration which may eventually lead to a reduction in diagnostic errors in clinical practice [14]. This trial aims to implement and evaluate this concept. To this end, existing data obtained in previous trials at UMG were analysed with regard to common clinical reasoning errors (CCRE) [15]. On this basis, e-seminars running in parallel to curricular teaching in the three aforementioned modules were modified in that – upon answering specific questions – students were prompted to comment on frequent CCREs (‘elaboration’). The analyses of student entries revealed that despite all the content having been covered in preceding teaching sessions, a considerable proportion of entries represented slack answers (e.g., ‘don’t know’ or ‘no idea’), suggesting that students might not have taken the exercise serious enough. In fact, this notion was corroborated in student comments during focus group discussions following the main study. As a consequence, the study was repeated in the following year, and this time complete answers to elaborations questions were incentivised using book vouchers. In this setting, a significant effect of the intervention was noted but student performance was still at best moderate. Given the importance of feedback for learning processes elicited by formative examinations [16], this aspect will be strengthened in the trial described here. Students can already open a text box containing static feedback after each question, but so far they have not received personal feedback after each exam. In winter term 2018/19, all students participating in the trial will receive individual emails containing (a) the raw point score achieved in each e-seminar, (b) static expert feedback to elaboration questions, and (c) their own entries to these elaboration questions. Thus, students will be able to compare their own answers to the instructor feedback.

## 3. Design and Conduct of the Study

This is a randomised controlled cross-over educational trial. Participating students will be stratified according to sex and summative exam scores in the previous term. Subsequently, they will be randomized to one of two study groups in a 1:1 fashion. During weekly e-seminars, they work on clinical cases addressing diagnostic and therapeutic strategies needed to manage patients with prevalent symptoms of general medical disorders. Cases will be presented as key feature cases with five questions per case. For some of these questions, elaboration questions will be written. These will focus on common misperceptions and clinical reasoning errors. When used as ‘intervention items’, elaboration questions will be shown after the original key feature question. Students will be prompted to enter a free-text answer. Upon completing both the original item and the elaboration question, they will be able to access a static feedback (‘expert comment’). This feedback will be included in an email sent to all students on the day after the e-seminar, also containing individual performance data as well as the student’s free-text answer to the elaboration question. When used as a ‘control item’, the same key feature question is being displayed, and students can access the expert comment directly after answering the question. Information on control items will not be contained in the mailed feedback. Every student will be exposed to 15 intervention and control items, respectively, and each of these will be shown twice over the course of 10 weeks. Items that are being shown as intervention items in one randomized group will be shown as control items in the other group and vice versa, thus making each student their own

control. At the end of the study, individual ‘intervention item’ and ‘control item’ scores will be computed for each student, and these two scores will be compared using a paired t Test. This primary analysis will be done to test the following hypothesis:

*„Long-term retention will be better for content that has been repeatedly tested with additional elaboration questions and subsequent mailed individual feedback than for content that has been repeatedly tested alone.“*

Long-term retention will be assessed in a formative electronic key feature assessment in summer term 2019. It will be identical to the entry and exit exam held in winter term 2018/19.

Secondary analyses will include unadjusted and adjusted linear regressions with percent scores in the exit exam and retention test as dependent variables and student characteristics as well as their engagement with key feature questions as independent variables.

## 4. Enrolment

Before the beginning of winter term 2018/19, all students enrolled to the three modules will receive an email describing the study. Particular emphasis will be put on the fact that study participation is voluntary. Students will be invited to participate in the trial. In this context, participation means that students agree to having their data analysed for the purpose of the trial. Every student will receive teaching and will also be invited to take e-seminars regardless of study participation. Written consent will be obtained in a classroom session on the first day of the module.

## 5. Archiving and Data Protection

For communication purposes, student names and email addresses need to be known. For each student providing written consent, these data as well as exam scores achieved in the preceding term will be provided by the Study Deanery. Personal data will be stored on a desktop computer to which only the Project Lead has access.

During e-seminars, students log onto the system using their individual credentials. Output files of the key feature examination system contain student login names. These will be used to merge files from various sources. Following the merging procedure and before running any analyses, all personal data will be deleted from the dataset. As a consequence, no individual student can be identified in this final dataset.

Responsibility for data treatment and storage lies with the Project Lead.

The Trial Protocol as well as all related documents have been approved by the institution’s data protection officer, Dr. Langbein.

Participating students are entitled to seek information on which of their personal data are being stored, and they can request all data to be deleted as long as they can still be identified in the dataset.

## 6. References


1. Raupach, T., et al., *Test-enhanced learning of clinical reasoning: a crossover randomised trial.* Med Educ, 2016. **50**(7): p. 711-20.
2. Raupach, T. and N. Schuelper, *Reconsidering the role of assessments in undergraduate medical education.* Med Educ, 2018. **52**(5): p. 464-466.
3. Ludwig, S., et al., *How can we teach medical students to choose wisely? A randomised controlled cross-over study of video- versus text-based case scenarios.* BMC Med, 2018. **16**(1): p. 107.
4. Wing, E.A., E.J. Marsh, and R. Cabeza, *Neural correlates of retrieval-based memory enhancement: an fMRI study of the testing effect.* Neuropsychologia, 2013. **51**(12): p. 2360-70.
5. Roediger, H.L. and J.D. Karpicke, *The Power of Testing Memory - Basic Research and Implications for Educational Practice.* Perspect Psychol Sci, 2006. **1**(3): p. 181-210.
6. Dobson, J.L. and T. Linderholm, *Self-testing promotes superior retention of anatomy and physiology information.* Adv Health Sci Educ Theory Pract, 2014.
7. Kromann, C.B., M.L. Jensen, and C. Ringsted, *The effect of testing on skills learning.* Med Educ, 2009. **43**(1): p. 21-7.
8. Kromann, C.B., et al., *The testing effect on skills learning might last 6 months.* Adv Health Sci Educ Theory Pract, 2010. **15**(3): p. 395-401.
9. Logan, J.M., A.J. Thompson, and D.W. Marshak, *Testing to enhance retention in human anatomy.* Anat Sci Educ, 2011. **4**(5): p. 243-8.
10. Baghdady, M., et al., *Test-enhanced learning and its effect on comprehension and diagnostic accuracy.* Med Educ, 2014. **48**(2): p. 181-8.
11. Larsen, D.P., A.C. Butler, and H.L. Roediger, 3rd, *Test-enhanced learning in medical education.* Med Educ, 2008. **42**(10): p. 959-66.
12. Page, G., G. Bordage, and T. Allen, *Developing key-feature problems and examinations to assess clinical decision-making skills.* Acad Med, 1995. **70**(3): p. 194-201.
13. Stark, R., V. Kopp, and M.R. Fischer, *Case-based learning with worked examples in complex domains: Two experimental studies in undergraduate medical education.* Learn Instruct, 2011. **21**: p. 22-33.
14. Norman, G.R. and K.W. Eva, *Diagnostic error and clinical reasoning.* Med Educ, 2010. **44**(1): p. 94-100.
15. Goldmann, M., et al., *[Choosing Wisely in medical education].* Z Evid Fortbild Qual Gesundhwes, 2017. **129**: p. 22-26.
16. Watling, C., et al., *Learning from clinical work: the roles of learning cues and credibility judgements.* Med Educ, 2012. **46**(2): p. 192-200.

## 7. Signature of the Project Lead

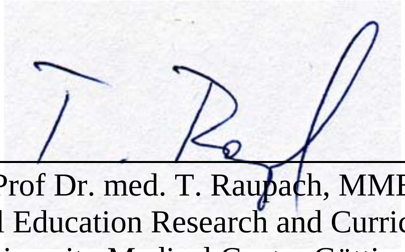

Prof Dr. med. T. Raupach, MME
Division of Medical Education Research and Curriculum Development
University Medical Centre Göttingen

## 8. Information for participants and informed consent

All students receive an electronic and a paper copy of the study information sheet. On the first day of the module, they will be invited to sign the participant consent form. By signing this form, students acknowledge that they will be enrolled in the trial. This decision can be revoked at any time during the trial.
