# Supplementary material for: Effects of Elaboration and Instructor Feedback on Retention of Clinical Reasoning Competence Among Undergraduate Medical Students: A Randomized Crossover Trial
Source: JAMA Netw Open. 2022 Dec 6;5(12):e2245491. doi: 10.1001/jamanetworkopen.2022.45491 (PMC9856325; doi:10.1001/jamanetworkopen.2022.45491)
Supplement: Supplement 2. — eFigure. CONSORT 2010 Flow Diagram eMethods. [file jamanetwopen-e2245491-s002.pdf]

Supplemental Online Content

Berens M, Becker T, Anders S, Sam AH, Raupach T. Effects of elaboration and instructor feedback on retention of clinical reasoning competence among undergraduate medical students: a randomized crossover trial. *JAMA Netw Open*. 2022;5(12):e2245491. doi:10.1001/jamanetworkopen.2022.45491

eFigure. CONSORT 2010 Flow Diagram
eMethods.

This supplemental material has been provided by the authors to give readers additional information about their work.

© 2022 Berens M et al. *JAMA Network Open.*

eFigure. CONSORT 2010 Flow Diagram

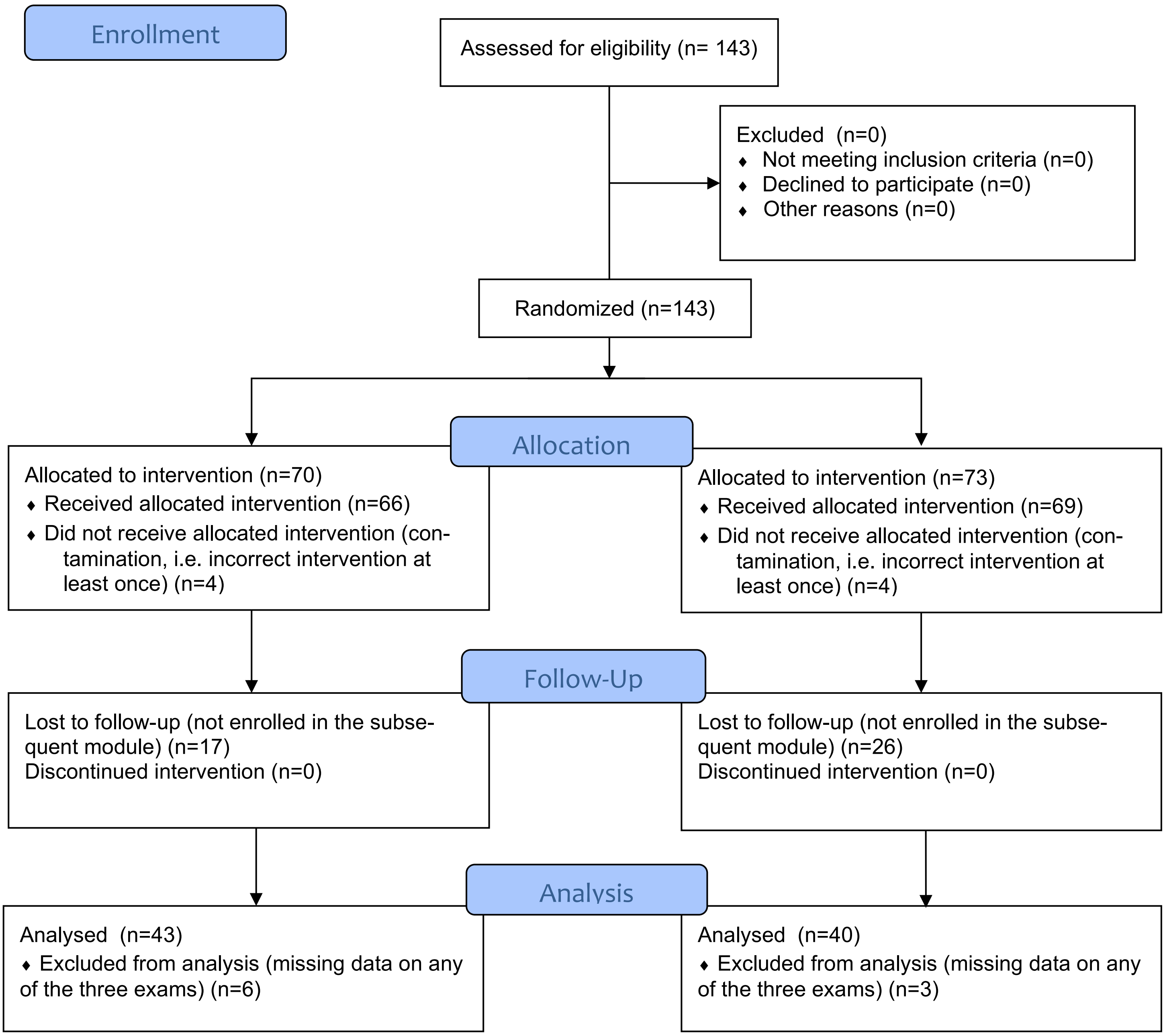

© 2022 Berens M et al. *JAMA Network Open.*

eMethods

Study design & Intervention

The trial was a randomised, controlled cross-over study with text-based key features only as the control condition. The study-related intervention took place between the entry and the exit exam and comprised the completion of ten weekly electronic seminars ('e-seminars'). In each of these 45-minute sessions, students gathered in the institution's computer resource room and completed four key feature cases with five long menu questions each. In this format, students are prompted to enter a few letters of their suggested answer, following which all options within the menu (approximately 4,000 in total) containing the letter string are displayed, and students can choose the answer they had been looking for.
Case content was aligned to module content of the preceding week, and some cases were designed to retrieve procedural knowledge that had been acquired more than a week ago. Key feature questions within these cases are referred to as 'items', and all analyses were done on the item level (and not on the level of complete cases). Whilst students were presented with a total of 20 key feature items every week (i.e., 200 items during the course of 10 e-seminars), only 30 of these served as 'intervention' and 'control' items within the context of this study; each item was either an intervention or a control item, depending on the group a particular student was allocated to (see below). Items were selected based on an analysis of e-seminar data obtained in previous student cohorts. They were characterised by the presence of at least one important incorrect answer that was chosen by a considerable number of students ('common clinical reasoning errors', CCRE). An example of a key feature question and an elaboration question addressing the respective CCRE is given in the **Table**. All items had undergone multiple review stages; in addition, all study-related procedures were established in a preceding pilot study.
The 30 intervention items were split in two sets of 15 and, stratified by previous item difficulties, randomly assigned to group A or group B, respectively. Students in group A were

exposed to 15 intervention items during the ten e-seminars while the other 15 items were presented without the elaboration question (‘control items’). All intervention and control items were shown twice over the course of 10 weeks in order to allow for repeated testing of the material. Students in group B received the opposite intervention, i.e. intervention items in this group corresponded to control items in group A. Each student enrolled served as their own control, i.e. every student was exposed to intervention as well as control items. Each of the 30 items was shown in the format of the intervention (with elaboration question) to one group and in the format of the control (without elaboration question) to the other group, respectively. Percent scores of intervention and control items answered correctly were calculated for each student.

Detailed feedback was created for each item, and all students were able to access the complete feedback in both the intervention and control format for any particular item. However, feedback texts were split up for intervention items: The first, shorter portion containing the correct answer to the key feature item was displayed before the elaboration question. The second portion containing an expert comment contrasting the correct answer with the CCRE was only displayed after the student had entered a free-text answer to the elaboration question. In the corresponding control item, the entire feedback was displayed at once. In addition, students received a partially automated but personalised email one day after each e-seminar, using a series letter function. This email contained the number of items answered correctly during that e-seminar as well as the mean and the range of raw points achieved by all students within that cohort. For each elaboration question answered by a student, the expert comment was included in the email, and students were also sent their original free-text answer to the corresponding elaboration question. They were encouraged to compare their own text to the expert comment. However, they did not receive an appraisal of the quality of their own response.

© 2022 Berens M et al. *JAMA Network Open.*

Measurement of clinical reasoning performance

Students sat three formative examinations: The entry exam was scheduled for the first day of the fourth year, and the exit exam was completed at the end of the first term. A retention test about six months after the exit exam served as the data collection point for the primary analysis.

All formative exams were made up of five key feature cases containing between five and eight questions (items) each for a total of 30 items. These tested the same content that was addressed in intervention items in e-seminars, but the case context was different. In order to ensure comparability, the same cases were used in the entry and exit exam as well as the retention test, but they were different from the cases presented in e-seminars occurring between the entry and exit exam. All exams were formative in nature, i.e. no incentive to achieve high scores was provided as this may have confounded results.

Student enrolment, data collection and analysis

Four weeks before the start of winter term 2018/19, students were informed about the study by e-mail. On the first day of term, they were invited to provide written consent to participate in the study, and consenting students were followed up over a total of nine months. In order to deliver the different interventions to students in groups A and B, every student was assigned a computer room where he/she would always receive the correct intervention for the respective e-seminar.

The primary outcome for this study was the within-subject difference in percent scores in intervention versus control items in the retention test six months after the last e-seminar (paired T test). Univariate and adjusted linear regression analyses were run using percent scores in the exit exam and the retention test as well as score differences between intervention and control items in these exams as the dependent variables as well as student age, sex, percent score in preceding written exams, and various descriptors of student

performance in e-seminars as the independent variables. Secondary outcomes included student performance in the exit exam and the proportion of correct answers as well as CCREs on individual items in e-seminars (first and second occurrence of an item) as well as in the exit exam and the retention test. In each of these cases, paired T tests were used to compare performance in intervention and control items, respectively.
