# Supplementary material for: Effects of Elaboration and Instructor Feedback on Retention of Clinical Reasoning Competence Among Undergraduate Medical Students: A Randomized Crossover Trial
Source: JAMA Netw Open. 2022 Dec 6;5(12):e2245491. doi: 10.1001/jamanetworkopen.2022.45491 (PMC9856325; doi:10.1001/jamanetworkopen.2022.45491)
Supplement: Supplement 3. — Data Sharing Statement [file jamanetwopen-e2245491-s003.pdf]

Data Sharing Statement

Berens. Effects of Elaboration and Instructor Feedback on Retention of Clinical Reasoning Competence Among Undergraduate Medical Students. *JAMA Netw Open*. Published December 06, 2022. doi:10.1001/jamanetworkopen.2022.45491

Data
Data available: No

Additional Information
Explanation for why data not available: Data sharing was not part of the original submission to the Ethics Committee, and participants have not provided written consent to having their data shared.
